# Physical comorbidity among patients attending mental health services at Windhoek Central Hospital, Namibia

**DOI:** 10.11604/pamj.2022.41.270.27134

**Published:** 2022-04-01

**Authors:** Ndahambelela Frederika Nepando Mthoko, Lilian Pazvakawambwa, Marja Leonhardt, Lars Lien

**Affiliations:** 1 Windhoek Central Hospital, Windhoek, Namibia,; 2 Department of Statistics and Population Studies, University of Namibia, Windhoek, Namibia,; 3 KoRus Øst, Hospital Innlandet Trust, PO Box 104, Brumunddal, Norway and Faculty of Health, VID-Specialized University, Oslo, Norway,; 4 Norwegian National Advisory Unit on Concurrent Substance Abuse and Mental Health Disorders, Hospital Innlandet Trust, PO Box 104, Brumunddal, Norway and Faculty of Social and Health Sciences, Inland Norway University of Applied Science, Elverum, Oslo, Norway

**Keywords:** Psychiatric conditions, physical conditions, co-morbid conditions

## Abstract

**Introduction:**

physical health problems are common among people with mental illness. Understanding common co-occurring mental and physical conditions can aid health providers to effectively screen individuals and also integrate care for both conditions. The study aimed to determine the prevalence and types of comorbidity among patients attending the outpatient section of the Mental Health Care Centre, Windhoek Central Hospital.

**Methods:**

a cross-sectional survey of 385 patients attending the Mental Health Care Centre of Windhoek Central Hospital was carried out using structured questionnaire.

**Results:**

the study found that 33.8% of participants had comorbid physical and mental conditions. The most common co-occurring physical conditions were from the cardiovascular system (40.8%), infections (30.8%), and neurological conditions (13.0%). Female patients were more likely to have comorbidity compared to their male counterparts (OR=2.8; CI = 1.5-5.0; p=0.001), and the risk of comorbidity increased with age (OR=1.1; CI = 1.0-1.1; p<0.001).

**Conclusion:**

the study emphasizes the inseparability of mental and physical health, and the bidirectional association between mental and physical conditions. The high prevalence of somatic disorder points to the need of integration of physical and mental health services. Mental health and somatic services must be adjusted to the fact that most of the people who come to seek help are likely to suffer from more than one illness.

## Introduction

There is an inborn inseparability of mind and body, of brain and thought, and of mental and physical health. Studies of community and clinic-based samples indicate that physical illness is often comorbid with mental illness [1]. Although the physical health of people with serious mental disorders (SMD), such as schizophrenia and other psychotic disorders, bipolar affective disorder, and moderate-to-severe depression is commonly ignored, they experience high rates of physical illness [2,3]. Globally, persons with SMD die about 10 to 25 years earlier than the general population [4,5]. Though there is some variation in the range of years of life lost, for example in Nordic countries, they die about 11 to 20 years earlier [6], and in Ethiopia they died about 30 years earlier than the general population [7], its extent appears consistent. This excess death is mostly from preventable physical diseases [8].

The most common co-morbid physical illnesses experienced by people with mental illness include cardiovascular diseases (CVD), diabetes, metabolic syndrome, respiratory illness and obesity related diseases [2]. Ofovwe CE and Ofovwe C [9] found people having HIV/AIDS to be more at risk of developing psychiatric comorbidities than the general population. The relationship between mental illness and HIV/AIDS is complex and bidirectional in nature. There is a greater risk of transmitting HIV if there are psychiatric co-morbidities [10]. According to Subedi *et al*. [10] such a risk is particularly great given the fact that individuals with prolonged psychiatric illnesses can exhibit poor judgment, affective instability and impulsivity.

Namibia is bearing the double burden of Communicable- and Non-Communicable Diseases (NCDs). In 2012 non-Communicable Diseases; such as CVD, stroke, Diabetes Mellitus, cancer and chronic respiratory diseases; and injuries were responsible for 43% of all deaths in Namibia [11]. According to the World Health Organization (WHO) NCDs Country Profiles for 2018 [12], CVD are the most common NCDs in Namibia, accounting for 17% of the total mortality. The leading cause of death in Namibia in 2013 was HIV/AIDS, causing over 20% of deaths [11]. Prevalence of HIV among adults aged 15-64 years in Namibia was 12.6% (15.7% among females and 9.3% among males) [13].

While country-level burden of physical conditions is known, estimates among the mentally ill are lacking. To reduce the mortality cap between those with and without mental disorders we need to learn more about the rate of somatic diseases among those attending mental health services. The objectives of this study was to determine the prevalence and the types of physical conditions among the patients attending Mental Health Care Centre (MHCC) and to explore the determinants of comorbidity.

## Methods

**Study design and setting:** this cross-sectional study was conducted between May and December 2017 at the outpatient of the MHCC, Windhoek Central Hospital.

**Participants:** the study population were service users who had mental illness and attended the outpatient of the MHCC. Inclusion criteria were both of the following: Adult patients attending the outpatient of the MHCC, aged 18 years and above, and those who could independently provide informed consent to participate in the study.

**Figure 1:**
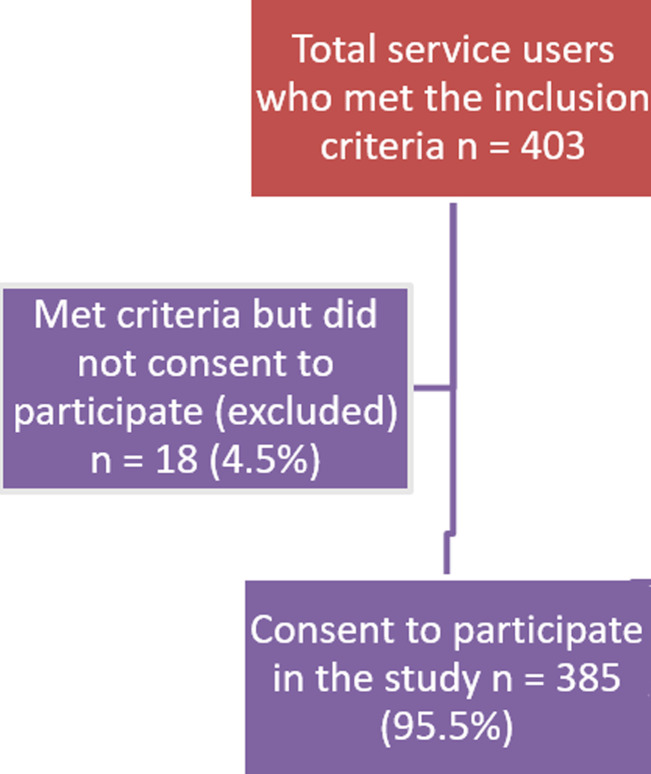
study flow chart

**Data collection:** a systematic random sampling method was used. Every fourth patient was given a number to take part in the study. In cases where the fourth patient was a child or did not have capacity to give consent, the subsequent patient was assessed. Medical officers at the MHCC, referred patients to researchers, after the routine assessment and management. Patients were sorted out for inclusion criteria; those who did not meet the criteria were thanked and excluded from the study. For those who were eligible and willing to participate in the study, explanations about the study were made, and an informed consent forms were signed. The data inclusion process is shown in Figure 1. Of the targeted sample size of 403; 385 (95.5%) accepted and participated in the study. Gender did not considerably influence refusal to participate as slightly more than half (56%) of those who refused to take part were males. Of the 18 (4.5%) who refused to take part in the study, some gave no particular reason for refusal, while others cited the following reasons: have to catch transport out of Windhoek; time; did not see the need; did not want; afraid; did not “feel like it”; and no benefit. Consented participants were then interviewed, by the researcher.

**Outcome measures:** study variables included gender, age at enrollment, education, employment, marital status, and socioeconomic status - low, medium, or high; where high socioeconomic status defined as having held a white collar/managerial job, medium socioeconomic status defined as other types of employment and low defined as unemployment. The outcome variable was comorbidity (whether or not a psychiatric patient had at least one co-occurring physical condition) Information about psychiatric and physical diagnoses, and the order of their development was obtained from both interviewing the participants and reviewing participant´s medical records.

**Data analysis and processing:** the data were analysed using the Statistical Package for Social Scientists (SPSS) version 23 statistical program. The dataset was coded before data entry and then assessed for errors and outliers. Descriptive summary statistics in the form of measures of centrality and dispersion and frequency distributions and charts were used to profile: 1. Background characteristics (age, sex, marital status, education, occupation, and socioeconomic status); 2. Psychiatric conditions (type of the psychiatric condition; whether the psychiatric condition was diagnosed before the physical condition; duration with the psychiatric condition; number of hospitalizations due to the psychiatric condition); 3. Physical conditions (type of physical condition; whether the physical condition was diagnosed before the mental condition) To establish whether there were significant associations between physical and mental conditions cross tabulations and Chi-squared tests of association was conducted. In all analyses, a probability value of p < 0.05 was considered statistically significant. A binary logistic regression model was fitted to establish the determinants of co-morbidity

**Ethics:** approval to carry out the study was obtained from the Ethics and Research Committee, Ministry of Health and Social Services, Windhoek, Namibia, and from the Norwegian regional ethic committee. Authorization of patients´ participation was also sought from the institution´s head. Participants were assured that the participation was completely voluntary, there would be no material gain from the study and they could withdraw from the study at any time in the course of the interview, and that refusal to participate would not in any way affect their health services/benefits which they were entitled to. All participants gave written consent to participate after the study procedures had been explained to them. To secure confidentiality serial numbers were assigned instead of names. The patients who did not consent to participate were excluded.

## Results

**Characteristics of the study participants:** the sociodemographic and clinical characteristics of the participants are presented in Table 1. The mean age at enrolment was 37.14 years and a standard deviation of 10.44 years. The majority of the participants were males (52.2%), middle aged (48.0%), single (79.0%), had secondary education (67.8%), unemployed (55.6%), and of low socioeconomic state (59.7%).

**Table 1:** sociodemographic and clinical characteristics of the participants

Characteristics of participants		Entire sample N=385	Participants with comorbidity N=130
**Sex**		n ( %)	n (%)
Female		184 (47.8)	77 (41.8)
Male		201 (52.2)	53 (26.4)
**Age**			
Youth (18-35 years)		180 (46.8)	40 (22.2)
Middle age (36 -55 years)		185 (48)	76 (41.1)
Older people (56 and above)		20 (5.2)	14 (70)
**Marital Status**			
	Single	304 (79.0)	88 (28.9)
	Married	58 (15.0)	26 (44.8)
	Separated	13 (3.4)	6 (46.1)
	Widowed	10 (2.6)	10 (100)
**Education**			
	None	7 (1.8)	4 (57.1)
	Primary	58 (15.1)	24 (41.4)
	Secondary	261(67.8)	90 (34.5)
	Tertiary	56 (14.5)	12 (21.4)
**Occupation**			
	Student	28 (7.3)	4 (14.3)
	Self-employed	25 (6.5)	10 (40.0)
	Employed	117 (30.4)	39 (33.3)
	Unemployed	214 (55.6)	77 (36)
**Socio-economic status**			
Low (never employed)		230 (59.7)	72 (31.3)
Medium (other employment)		127 (33.0)	50 (39.4)
High (white collar job)		28 (7.3)	8 (28.6)
**Psychiatric conditions**			
Schizophrenia spectrum and other psychotic disorders		210(54.5)	62(29.5)
Bipolar and related disorders		68(17.7)	23 (33.8)
Depressive disorder		86(22.3)	31 (36.0)
Anxiety disorders		7(1.8)	5(71.4)
Trauma and stress or related disorders		9(2.3)	5 (55.5)
Somatic Symptom and related disorders		3(0.8)	2(66.7)
Sleep- wake disorders		2(0.5)	2 (100)

Psychiatric conditions among the participants included schizophrenia (30%), major depressive disorder (20%), bipolar I disorder (16.9%), alcohol induced psychotic disorder (6.5%) and psychotic disorder due to another medical condition (5.5%). The duration with the psychiatric condition ranged from 0 to 41 years with a mean of 7.27 years and a standard deviation of 7.5 years. The number of hospitalizations with the psychiatric condition ranged from 0 to 24 times with a mean of 2.38 and standard deviation of 2.5 times.

Co-morbid conditions: hundred and thirty (33.8%) of the participants had physical conditions comorbid with psychiatric conditions. More females 77(59.2%) were having comorbidity compared to males 53(40.8%). There was a significant relationship between comorbid condition and demographic characteristics (age group (χ^2^=27.324, p<0.001); marital status (χ^2^=26.837, p<0.001); occupation (χ^2^=11.087, p=0.026). The level of education (χ^2^=7.046, p<0.070) and the socioeconomic status (χ^2^=1.611, p<0.447) of the patient were not significantly associated with comorbid condition. Among females, there were significant association between psychiatric conditions and physical conditions (χ^2^ =99.932, P< 0.001), while among the males the relationship between psychiatric conditions and physical conditions was not significant (χ^2^ =20804, P = 0.289).

The most common comorbid were co-morbidities between psychiatric disorders and conditions of cardiovascular system, infections and neurological conditions (40.8%, 30.8%, and 13.0% respectively among participants with comorbidity, which is 13.8%, 10.4%, 4.4% respectively among all participants). Among those with co-occurring psychiatric conditions and infections, 27.0% were due to HIV.

Schizophrenia spectrum disorders; bipolar and related disorders; depressive disorders; and anxiety disorders tend to co-occur with conditions of cardiovascular system and infections. Psychiatric conditions likely to comorbid with HIV were anxiety (40%), schizophrenia spectrum and other psychotic disorders (32.3%), bipolar and related disorders (30.4%), and depressive disorders (22.6%). There was a significant association between the patient´s psychiatric condition and their physical condition (χ^2^ = 135.822, p<0.001). Table 2 shows the association between comorbid conditions.

**Table 2:** association between psychiatric and physical conditions among participants with comorbid conditions

Psychiatric conditions	Physical conditions					Total comorbidity N(%)
	Cardiovascular system N (%)	Infections N (%)	Neurological N (%)	Endocrine metabolic N (%)	Others N (%)	
Schizophrenia Spectrum and Other Psychotic Disorders	23 (37.1)	22 (35.5)	13 (21.0%)	1(1.6)	3(4.8)	62(47.7)
Bipolar and Related Disorders	10 (43.5)	8 (34.8)	1 (4.3)	2(8.7)	2(8.7)	23(17.7)
Depressive Disorder	15 (48.4)	8(25.8)	2(6.4)	2(6.4)	4(12.9)	31 (23.8)
Others	5(35.7)	2 (14.3)	1 (7.1)	2(14.3)	4 (28.6)	14(10.8)
Total	53 (40.8)	40(30.8)	17 (13.0)	7(5.4)	13(10.0)	130

**Figure 2:**
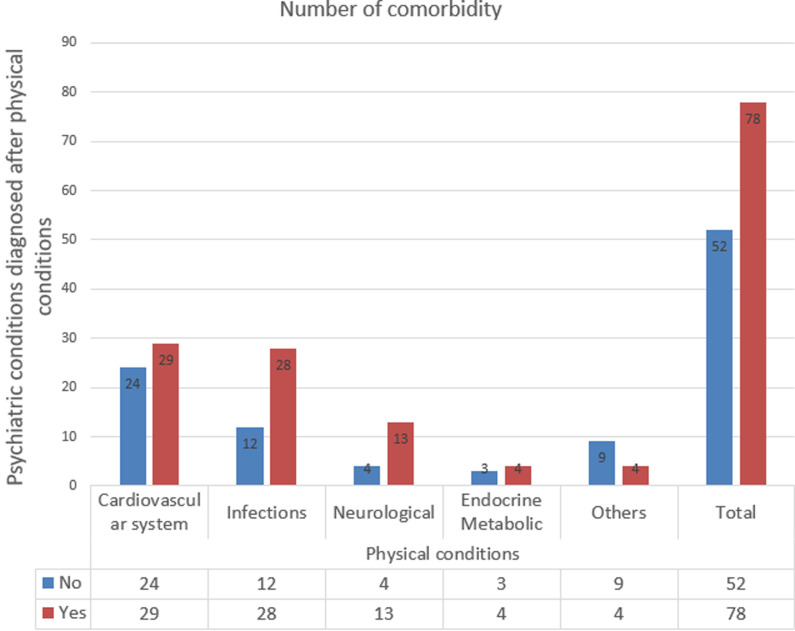
sequence of comorbidity

Among those with comorbid conditions, 91.5% had one comorbid condition, 6.9% had two and 0.7% had three comorbid conditions. Sequence of development of comorbid conditions: Out of the 130 participants with comorbidity, 78 (60%) developed psychiatric conditions after the onset of the physical conditions, that is 20.3% of all the participants. Thirty-five (16.7%) of those with schizophrenia spectrum and other psychotic disorders, 21 (24.4%) of those with depressive disorders and 8 (11.8%) of those with bipolar and related disorders developed their conditions after physical conditions. The average period or years up to or until the onset of psychiatric condition was 8.2 years (95% confidence Interval (CI) = (6.19 -10.24) years). Out of 78 participants who developed physical conditions first, 29 (37.2%) had conditions of cardiovascular system and 28 (35.9%) had infections. Figure 2 shows the sequence of development of comorbidity.

Determinants of comorbidity: the binary logistic regression results to establish the determinants of comorbidity are shown in Table 3. Comorbidity was associated with sex, age, education, occupation and socioeconomic status of the patient. Females were more likely to have comorbidity compared to their male counterparts (OR=2.062: 95% CI =1.271-3.346, p=0.003). The risk of comorbidity increased with age (OR=1.028: 95% CI = 1.005-1.052, p=0.016). The risk of comorbidity decreased with increasing level of education. Patients with no formal education were more likely to have comorbidity compared to their counterparts with tertiary education (OR=7.963: 95% CI 1.307-48.539, p=0.024). With regard to occupation, self-employed patients were less likely to have comorbidity compared to those employed (OR=0.187: 95% CI 0.073-0.476, p<0.001). Patients with low socioeconomic status (never employed) were less likely to have comorbidity compared to those with high socioeconomic status (white collar job) (OR=0.065: 95% CI 0.011-0.367, p=0.002).

**Table 3:** risk factors for comorbidity

Independent Variable	p-value	Odds Ratio	95% C.I.for Odds Ratio	
			Lower	Upper
**Sex**	**.003**	2.062	1.271	
Female				3.346
Male (Ref)				
**Age**	**.016**	1.028	1.005	1.052
**Education**				
None	**.024**	7.963	1.307	48.539
Primary	.072	2.550	.921	7.065
Secondary	.086	2.052	.902	4.668
Tertiary (Ref)				
**Marital Status**				
Single, Separated or Widowed	.687	.880	.473	1.638
Married (Ref)				
**Occupation**				
Student	.542	.559	.086	3.634
Self Employed	**<0.001**	.187	.073	.476
Unemployed	.995	1.005	.204	4.941
Other	.947	1.063	.173	6.551
Employed (Ref)				
**Socioeconomic Status**				
Low (Never Employed)	**.002**	.065	.011	.367
Medium (Other Employment)	.055	.362	.128	1.021
High (White Collar job) Ref				

## Discussion

One third of the patients receiving mental health services from the Windhoek Central Hospital had co-occurring somatic conditions The most common comorbidity was conditions of the cardiovascular system, followed by infections and neurological conditions. About 20% of the participants developed psychiatric conditions after their physical conditions. The female and elderly were more prone to have co-occurring conditions.

This is the first study in the country to report on comorbid between mental and physical conditions. The real number of comorbidity might be higher than 33.8%, because the study population consisted only of those who were able to give consent, who might be healthier than other patients at the hospital. Although the results are not broadly generalizable, we believe the study results have relevance to the countries similar to Namibia in infrastructure and development of mental health care. The high prevalence of comorbidities could be attributable to the current epidemiological transition in the country with an ongoing increase in non-communicable diseases. The double burden of Non-Communicable and Communicable disease to Namibia was also evident in the case of comorbid of mental and physical conditions, where 40.8% were due to conditions of cardiovascular system and 30.8% were due to infections.

Among the participants with comorbid conditions, 40.8% were due to conditions of cardiovascular system, while 36% of the participants with depressive disorders had comorbid conditions. The findings are similar to the study by O´Sullivan *et al*. [2] that found common comorbidities to be that of psychiatric condition and cardiovascular diseases and Naylor *et al*. [14] who reported that the risk of physical illness was high among people with a diagnosis of severe mental illness. Fagiolini *et al*. [15] demonstrated that people with bipolar disorder also share the excess burden of cardiovascular problems, which is the same case in the current study were among participants who had Bipolar and related disorders comorbid with physical conditions, 43.5% were with conditions of cardiovascular system. Several factors have been found to contribute to the high prevalence of physical conditions among people with severe mental illness and that include sedentary behaviour, treatment with antipsychotic medication, unequal access to health care [16-18] and the consequences of mental illness.

People with physical health problems are at increased risk of poor mental health. The study showed that not only mental disorders could precede the onset of physical disorders, but also that physical disorders could precede the onset of mental disorders. Of the participants, 20.3% had physical conditions that precede the development of psychiatric conditions, while 13.5% had the psychiatric conditions that precede physical conditions. This emphasize that poor physical health can lead to an increased risk of developing mental health problems. Again conditions of the cardiovascular system (37.2%) and infections (35.9%) precede the development of psychiatric conditions. Prospective tracking of cross-disorder morbidity will be important to establish mechanisms for prevention and intervention.

Females and the elderly were at high risk of comorbidity. The successes of medicine in prolonging life without curing disease and makes it easier to simultaneously contract two or more illnesses and the high average life expectancy among females could be possibly the contributing factor.

The additive effects of co-occurring physical and mental conditions often produce worse overall health outcome [19]. Persons with SMD die about 10-25 years earlier than the general population. The majority of deaths are due to cardiovascular disease, respiratory disease, and infections [20,21]. The co-occurrence of SMD and medical comorbidity increased the complexity of the person´s needs and frequently created difficulties with self-management [22], capacity to navigate services, ability to undertake preventive measures, and the likelihood of following-up on treatment recommendations [23]. Review articles [23,24] provide confirmation that the simultaneous presence of two or more diseases makes the treatment of all of them more difficult, leading to increasing number of complications, and worsens the prognosis of all the diseases that are present.

## Conclusion

The study examined the prevalence of co-occurrence of physical conditions with SMD and the possible determinants of comorbidity. The study emphasizes the inseparability of mental and physical health, and the bidirectional association between mental and physical conditions. People with mental disorders are at increased risk of physical conditions comorbid to their illness and people with physical conditions are at risk of developing mental illness. The high prevalence of comorbidity points to the need to integrate physical and mental health services. Health services will have to adjust to the fact that most of the people who come to seek help are likely to suffer from more than one illness.

### What is known about this topic

- *Mental health problems often co-exist with physical health problems;*
- *The common comorbid is that of mental illness and conditions of cardiovascular system*.

### What this study adds

- *Among people with comorbid conditions, the psychiatric conditions mostly developed after the physical conditions;*
- *Provides important and not previously available information about the prevalence and pattern of co-occurrence mental disorders and physical disorders in developing countries*.
